# Galangin Exhibits Neuroprotective Effects in 6-OHDA-Induced Models of Parkinson’s Disease via the Nrf2/Keap1 Pathway

**DOI:** 10.3390/ph15081014

**Published:** 2022-08-17

**Authors:** Qiu-Xu Chen, Ling Zhou, Tao Long, Da-Lian Qin, Yi-Ling Wang, Yun Ye, Xiao-Gang Zhou, Jian-Ming Wu, An-Guo Wu

**Affiliations:** 1 Sichuan Key Medical Laboratory of New Drug Discovery and Druggability Evaluation, Materia Medica, Luzhou Key Laboratory of Activity Screening and Druggability Evaluation for Chinese Materia Medica, School of Pharmacy, Southwest Medical University, Luzhou 646000, China; 2 Department of Pharmacy, Affiliated Hospital of Southwest Medical University, Luzhou 646000, China; 3 Education Ministry Key Laboratory of Medical Electrophysiology, School of Preclinical Medicine, Southwest Medical University, Luzhou 646000, China

**Keywords:** galangin, 6-OHDA, Parkinson’s disease, Keap1/Nrf2, network pharmacology

## Abstract

Parkinson’s disease (PD) is the second most common neurodegenerative disease, and there is still no cure for it. PD is characterized by the degeneration of dopaminergic neurons, and oxidative stress has been considered an important pathological mechanism. Therefore, the discovery of antioxidants to alleviate the oxidative damage of dopaminergic neurons is a promising therapeutic strategy for PD. First, a network pharmacology approach was used, and nine common core targets of galangin and PD were screened, mainly involving cell aging, apoptosis, and cellular responses to hydrogen peroxide and hypoxia. In addition, the Gene Ontology (GO) function and pathway enrichment analysis of the Kyoto Encyclopedia of Genes and Genomes (KEGG) identified apoptosis, PI3K/Akt, and HIF-1 signaling pathways. Furthermore, the molecular docking results revealed a strong affinity between galangin and the NFE2L2/Nrf2 protein. To validate the above predictions, we employed 6-hydroxydopamine (6-OHDA) to induce neuronal death in HT22 cells and *Caenorhabditis elegans* (*C. elegans*). MTT, cell morphology observation, and Hoechst 33342-PI staining results showed that galangin significantly increased the viability of 6-OHDA-treated HT22 cells. In addition, galangin inhibited 6-OHDA-induced ROS generation and apoptosis in HT22 cells. Mechanistic studies demonstrated that galangin activates the Nrf2/Keap1 signaling pathway, as evidenced by the decreased protein expression of Keap1 and increased protein expression of Nrf2 and HO-1. In the 6-OHDA-induced PD model of *C. elegans*, galangin indeed inhibited the degeneration of dopaminergic neurons, improved behavioral ability, and decreased ROS generation. In conclusion, the current study is the first to show that galangin has the capacity to inhibit neuronal degeneration via the Nrf2/Keap1 pathway, suggesting that galangin is a possible PD treatment.

## 1. Introduction

Parkinson’s disease (PD) is the second most common neurodegenerative disease with an overall prevalence of 0.3%, and it has risen to 3.5% in people aged 85–89, which places a heavy burden on our economy and society [1]. The degeneration of dopaminergic neurons in the substantia nigra and the formation of Lewy bodies are the main pathological features of PD [2]. Although current drugs have the ability to ameliorate the clinical symptoms of PD, there are still no drugs that can slow the progression of the disease [3]. Therefore, the discovery of effective drugs for the treatment of PD has been a research hot spot in future studies.

Under normal physiological conditions, the redox state between oxidants (or pro-oxidants) and antioxidants is balanced. However, when this balance is disrupted, excessive ROS are generated and induce oxidative damage to targeted cells or tissues. It is believed that oxidative damage is a key factor that leads to the loss of nigrostriatal dopaminergic neurons in PD subjects [4]. The excessive generation of ROS, in turn, further causes mitochondrial dysfunction and aggravates the progression of PD [5]. The autopsy reports showed that oxidative damage could be found in the cerebral substantia nigra of PD patients, which is evidenced by the significantly elevated levels of free iron ions [6], reduced levels of glutathione [7], and numerous oxidized lipids, proteins, and DNA. Nrf2, the homolog of the hematopoietic transcription factor p45 NF-E2, plays an important role in countering oxidative stress or activating the antioxidant response by inducing the expression of various transcription factors, such as HO-1, NQO1, and GCLC [8,9]. Emerging evidence indicates that the activation of the Nrf2/Keap1 signaling pathway can protect against oxidative damage induced by misfolded protein aggregates and toxins associated with PD [9]. Therefore, the identification of Nrf2 activators is a promising strategy to protect dopaminergic neurons against oxidative damage in PD.

Galangin is a flavonol belonging to the flavonoid family, with high contents in plants such as *Alpinia officinarum*, *Helichrysum aureonitens*, and *Alpinia galanga* and propolis [10,11]. Numerous studies have shown that galangin exhibits multiple pharmacological activities, such as anti-mutagenic, anti-carcinogenic, antioxidant, and anticancer effects [12]. However, its neuroprotective effects on PD and the related mechanisms of action are still unknown. In this study, the potential targets and signaling pathways of galangin in PD were predicted by network pharmacology and a molecular docking approach [13]. Then, we investigated the neuroprotective effect of galangin in 6-OHDA-treated HT22 cells and BZ555 worms and found that galangin significantly increased the viability of 6-OHDA-treated HT22 cells by reducing the generation of intracellular ROS levels and inhibiting cell apoptosis. A mechanistic study revealed that galangin activated the Nrf2/Keap1 signaling pathway. Moreover, the neuroprotective effect of galangin was validated in 6-OHDA-treated *Caenorhabditis elegans* (*C. elegans*). Therefore, the findings in this study provide novel insights into galangin as a candidate for PD for the first time in the future.

## 2. Results

### 2.1. Identification of Potential Therapeutic Targets

**Figure 1:**
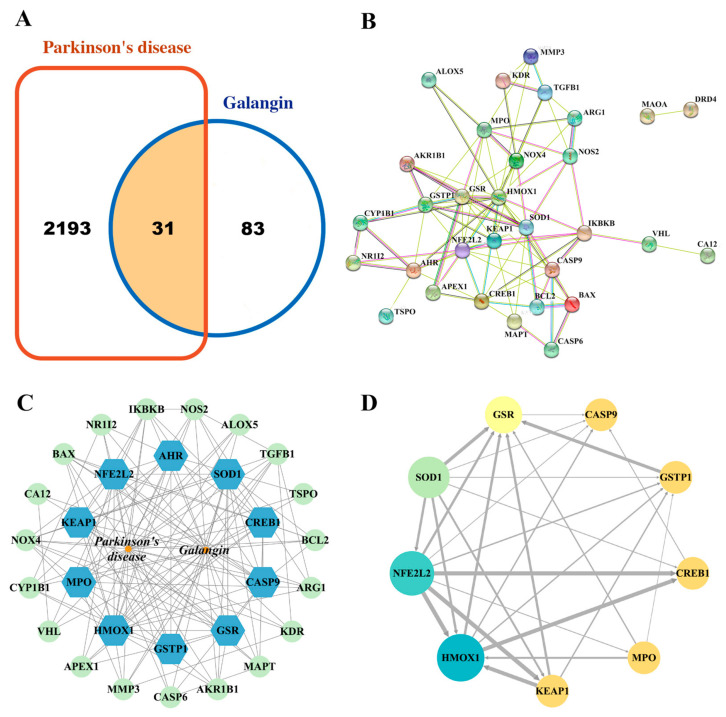
Network pharmacology analysis of galangin in PD. (**A**) Venn diagram of the common targets of galangin in PD. (**B**) The potential therapeutic targets were analyzed using the STRING database. (**C**) The construction of the galangin-target-PD network by the Cytoscape v3.7.1 software (Cytoscape Consortium, Seattle, WA, USA). (**D**) Protein–protein interaction (PPI) network based on the core targets of galangin against PD according to the screening conditions of Degree > 8, BC > 0.01815109, and CC > 0.5.

The potential targets of galangin for the treatment of PD were predicted using network pharmacology. The “drug targets” and “disease targets” were integrated through screening using databases. A total of 114 targets were found to be linked to galangin based on the results of data integration using the SwissTargetPrediction, GeneCards, and PharmMapper databases. After the deletion of duplicates, 2224 targets connected to PD were retrieved from the DisGeNET and OMIM databases. Among them, 31 common targets were considered potential targets of galangin for the treatment of PD (Figure 1A).

### 2.2. Topology Analysis of the PPI Network and Acquisition of Core Targets

The 31 potential therapeutic targets identified above were further analyzed by the STRING database. The data were imported into Cytoscape v3.7.1 software (Cytoscape Consortium, Seattle, WA, USA) to generate an interaction network and nine core targets, including HMOX1, NFE2L2, SOD1, GSR, CASP9, KEAP1, MPO, GSTP1, and CREB1 were obtained using a topology analysis (Figure 1B–D).

### 2.3. Enrichment Analysis of the Core Targets of Galangin in PD

**Figure 2:**
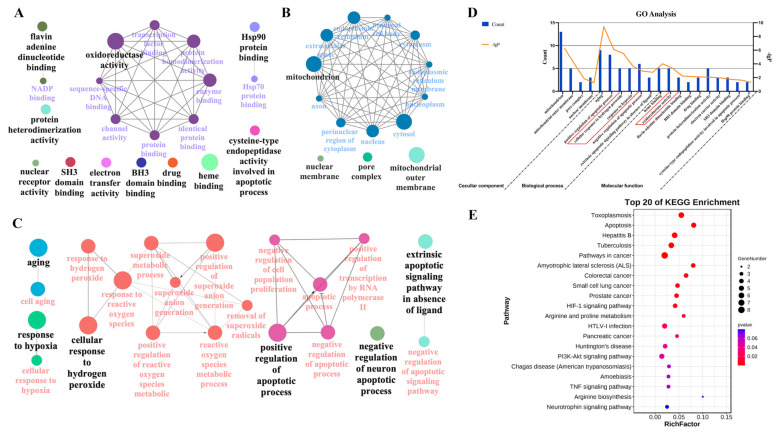
GO and KEGG enrichment analyses of galangin in the treatment of PD. (**A**) Visualization of 20 terms of molecular function enrichment analysis. (**B**) Visualization of 14 terms of cellular component enrichment analysis. (**C**) Visualization of 21 terms of biological process enrichment analysis. (**D**) GO analysis of core targets of galangin in the treatment of PD. Four terms of CC, 6 terms of BP, and 10 terms of MF showing the greatest enrichment of galangin-related processes are listed according to *p* values. (**E**) The top 20 KEGG pathways enrichment analysis involved in the potential treatment of galangin in PD.

The potential signaling pathways regulated by galangin in PD were elucidated by GO and KEGG enrichment analyses. The GO analysis was performed through the DAVID platform, and the results were visualized by Cytoscape plug-in ClueGO_v3.9.0 software (Cytoscape Consortium, Seattle, WA, USA). The colors, nodes, and the lines between nodes represent different clustering functional groups, enrichment pathways, and numbers of genes shared between pathways, respectively. As shown in Figure 2A–C, a total of 55 GO terms were related to PD and galangin. Among them, 21 terms linked to the BP category mainly involve cell aging, apoptotic process, oxidative stress, and hypoxia; 14 terms related to the CC category mainly involve the mitochondria, mitochondrial outer membrane, pore complex, and nuclear membrane; the 20 terms associated with the MF category mainly involve heme binding, oxidoreductase activity, flavin adenine dinucleotide binding, and BH3 domain binding. Finally, six BP terms, four CC terms, and 10 MF terms showed the greatest enrichment with galangin-related processes according to *p* values (Figure 2D). In addition, the KEGG enrichment analysis revealed that the regulation of galangin on the signaling pathways in PD was significantly enriched in apoptosis, HIF-1, TNF-α, and neurotrophic-related pathways. As shown in Figure 2E, the 20 most-enriched pathways were arranged according to *p* values.

### 2.4. Molecular Docking Assessment

**Figure 3:**
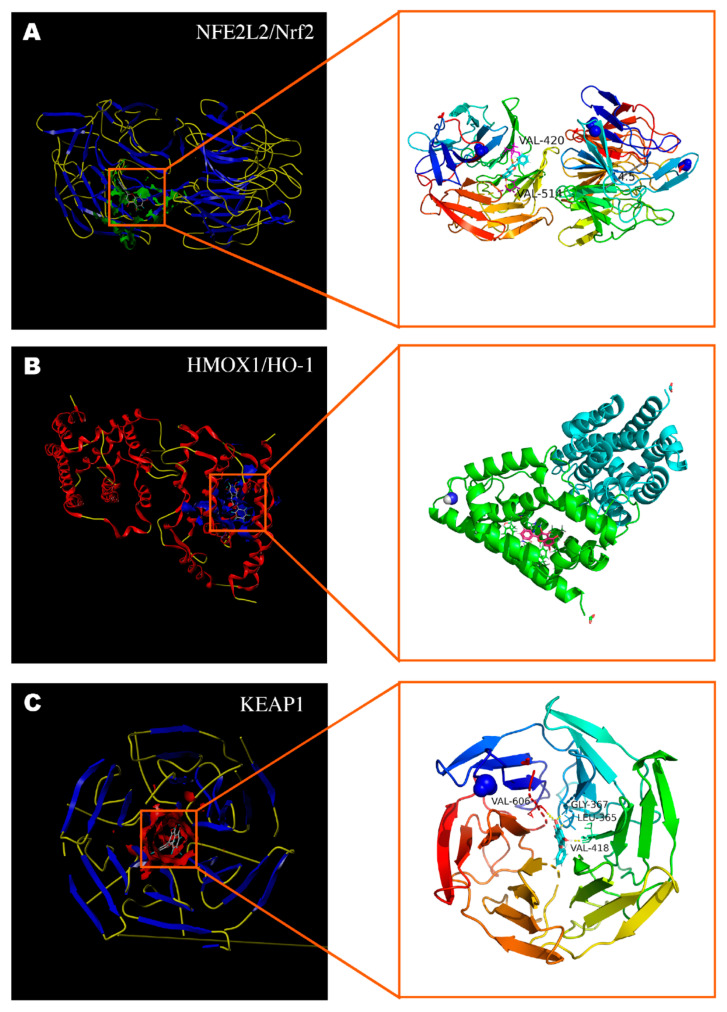
Molecular docking of galangin with core targets. (**A**) Galangin binds to NFE2L2/Nrf2 protein (7K29) via the active residues VAL-420 and VAL-514. (**B**) Galangin binds to HMOX-1/HO-1 protein (6EHA). (**C**) Galangin binds to Keap1 protein (1ZGK) via the active residues VAL-418, VAL-606, GLY-367, and LEU-365.

To further explore the direct relationship between galangin and the core targets, we employed molecular docking simulations to estimate their binding ability. Figure 3 displays the docking of galangin with Nrf2, HO-1, and Keap1, and the docking scores are 5.52, 4.80, and 4.63, respectively, which indicates that there is a high binding strength of galangin with Nrf2 [14,15]. Therefore, galangin may regulate the Nrf2/Keap1/HO-1 signaling pathway.

### 2.5. Galangin Decreases Cell Death in 6-OHDA-Treated HT22 Cells

**Figure 4:**
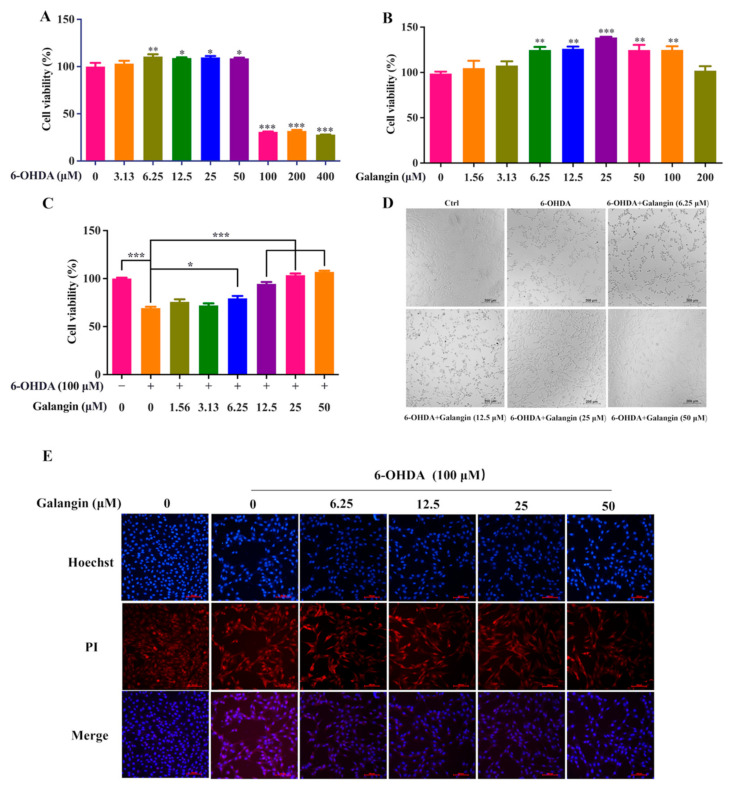
Galangin decreased cell death in 6-OHDA-treated HT22 cells. (**A**) MTT assay of the viability of HT22 cells treated with 3.13–400 μM 6-OHDA for 24 h. (**B**) MTT assay of the viability of HT22 cells treated with 1.56–200 μM galangin for 24 h. (**C**) MTT assay of the viability of HT22 cells treated with 1.56–50 μM galangin in the absence or presence of 6-OHDA for 24 h. (**D**) Representative images of the cell morphology of HT22 cells treated with 6.25–50 μM galangin in the absence or presence of 6-OHDA for 24 h. Magnification: 10×; Scale bars: 200 µm. (**E**) Representative Hoechst/PI staining images of HT22 cells treated with 6.25–50 μM galangin in the absence or presence of 6-OHDA. Magnification: 10×; Scale bars: 100 µm. Bars, S.D., * *p* ≤ 0.05, ** *p* ≤ 0.01, *** *p* ≤ 0.001.

Emerging evidence indicates that 6-OHDA, a PD toxin, is commonly used to induce cellular and animal models of PD [16]. Therefore, we employed 6-OHDA to induce cell death in HT22 cells. First, we measured the cytotoxicity of 6-OHDA and galangin in HT22 cells using the MTT assay, as shown in Figure 4A. The treatment with 100–400 μM 6-OHDA significantly decreased the viability of the HT22 cells. However, 1.56–200 μM galangin showed no cytotoxicity in HT22 cells (Figure 4B). Then, we investigated the neuroprotective effect of galangin in 6-OHDA-treated HT22 cells. HT22 cells were pretreated with a series of concentrations of galangin for 24 h and then exposed to 100 μM 6-OHDA for another 24 h. Figure 4C shows that galangin significantly increased the viability of 6-OHDA-treated HT22 cells. Additionally, the morphology of 6-OHDA-treated HT22 cells was indeed improved by galangin (Figure 4D). Moreover, the Hoechst/PI staining results showed that galangin significantly decreased the cell death of 6-OHDA-treated HT22 cells, as demonstrated by the decreased number of cells with PI/Hoechst signals (Figure 4E). Taken together, galangin has the capacity to restore the viability of the 6-OHDA-treated HT22 cells.

### 2.6. Galangin Inhibits ROS Production and Cell Apoptosis in 6-OHDA-Treated HT22 Cells

**Figure 5:**
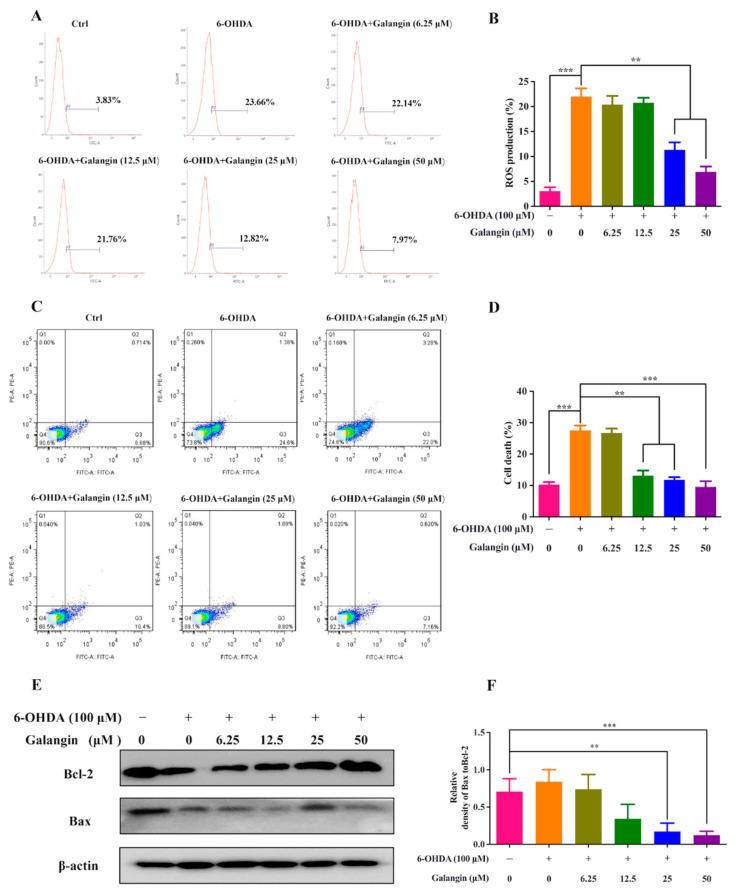
Galangin reduced 6-OHDA-induced intracellular ROS generation and apoptosis in HT22 cells. (**A**) HT22 cells were treated with galangin in the absence or presence of 6-OHDA at the indicated concentrations for 24 h. After treatment, the cells were incubated with 5 μM H2DCFDA probe for 30 min and subjected to flow cytometry analysis. (**B**) Bar chart indicates the percentage of HT22 cells with H2DCFDA signals. Bars, S.D., ** *p* ≤ 0.01, *** *p* ≤ 0.001. (**C**) HT22 cells were treated with galangin in the absence or presence of 6-OHDA at the indicated concentrations for 24 h. After treatment, the cells were collected and analyzed by flow cytometry using an Annexin V-FITC apoptosis detection kit according to the manufacturer’s instructions. (**D**) Bar chart indicates the apoptosis rate of HT22 cells. Bars, S.D., ** *p* ≤ 0.01, *** *p* ≤ 0.001. (**E**) HT22 cells were treated with galangin in the absence or presence of 6-OHDA at the indicated concentrations for 24 h. After treatment, cell lysates were then collected for the protein detection of Bax, Bcl-2, and β-actin by Western blot. (**F**) Bar chart indicates the ratio of Bax/Bcl-2. Bars, S.D., ** *p* ≤ 0.01, *** *p* ≤ 0.001. The full-length Western blotting images are shown in Supplementary Figure S1.

Midbrain dopaminergic neurons are sensitive to oxidative stress, and 6-OHDA has been demonstrated to induce the generation of ROS and neuronal apoptosis [17]. Therefore, we further investigated whether galangin inhibited ROS production and cell apoptosis in 6-OHDA-treated HT22 cells. The flow cytometry analysis results showed that galangin dose-dependently decreased ROS levels induced by 6-OHDA (Figure 5A,B). In addition, the apoptosis rate of the HT22 cells detected by flow cytometry was significantly inhibited by galangin in a dose-dependent manner (Figure 5C,D). Moreover, the ratio of protein expression of Bax to Bcl-2 was reduced by galangin (Figure 5E,F). Taken together, galangin decreased the cell death of the 6-OHDA-treated HT22 cells by inhibiting intracellular ROS production and cell apoptosis.

### 2.7. Galangin Activates the Nrf2/Keap1/HO-1 Signaling Pathway

**Figure 6:**
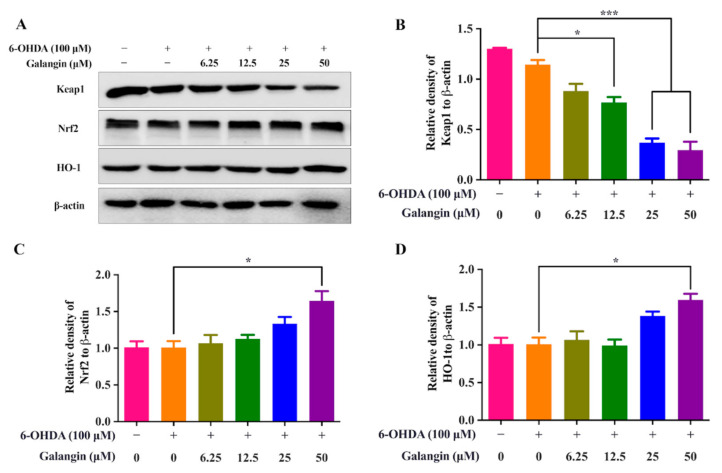
Galangin activates the Keap1/Nrf2/HO-1 signaling pathway. (**A**) HT22 cells were treated with galangin in the absence or presence of 6-OHDA at the indicated concentrations for 24 h. After treatment, cell lysates were collected and harvested for the protein detection of Keap1, Nrf2, HO-1, and β-actin by western blot. (**B**) The bar chart indicates the relative density of Keap1 to β-actin. (**C**) The bar chart indicates the relative density of Nrf2 to β-actin. (**D**) The bar chart indicates the relative density of HO-1 to β-actin. Bars, S.D., * *p* ≤ 0.05, *** *p* ≤ 0.001. The full-length Western blotting images are shown in Supplementary Figure S1.

Numerous studies have shown that the Keap1-Nrf2/ARE signaling pathway is the most important endogenous antioxidant signaling pathway [18]. Under the conditions of oxidative stress or treatment with Nrf2 activators, Nrf2 dissociates from Keap1 and is translocated into the nucleus to initiate the expression of various downstream target proteins, such as HO-1 [19,20]. In this study, the protein expression of Keap1, Nrf2, and HO-1 in HT22 cells was examined by Western blotting. The results showed that galangin significantly inhibited the protein expression of Keap1 while increasing the protein expression of Nrf2 and HO-1 (Figure 6), suggesting that galangin activated the Keap1/Nrf2/HO-1 signaling pathway. Therefore, galangin inhibits 6-OHDA-induced oxidative damage via the Keap1/Nrf2/HO-1 signaling pathway.

### 2.8. Galangin Exhibits Neuroprotective Effects in 6-OHDA-Treated BZ555 Worms

**Figure 7:**
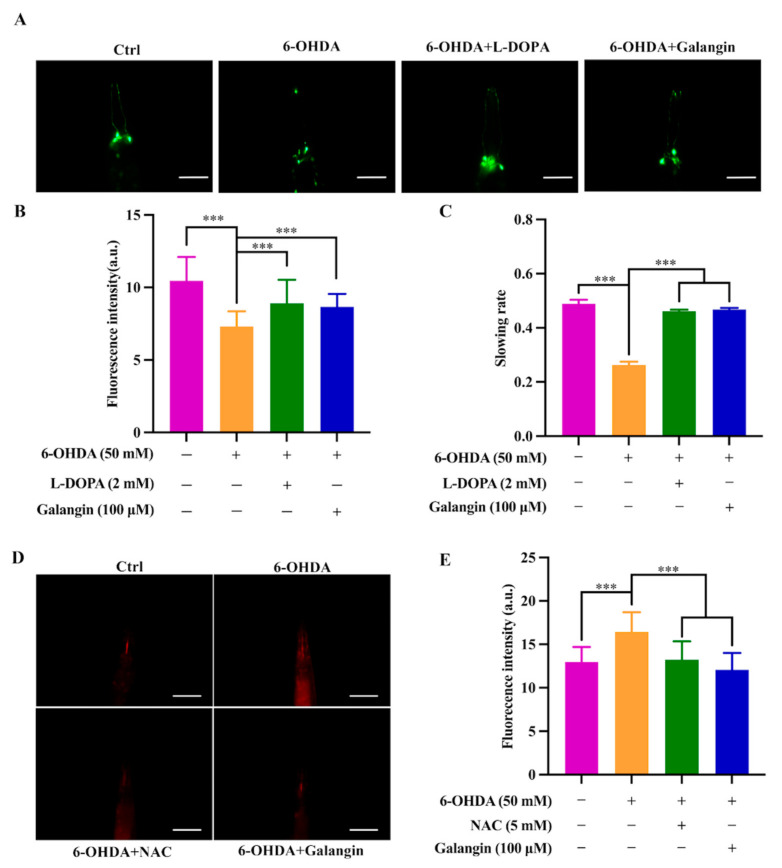
Galangin exerts a neuroprotective effect in 6-OHDA-treated BZ555 worms. (**A**) Representative images of BZ555 worms treated with galangin (100 μM) or L-Dopa (2 mM) in the presence or absence of 6-OHDA (50 mM) for 72 h. The GFP represents the viability of DA neurons. Magnification: Scale bars: 20 µm. (**B**) The bar chart indicates the quantification of GFP intensity in BZ555 worms. Bars, S.D., *** *p* ≤ 0.001. (**C**) The bar chart indicates the relative slowing rate (%) of BZ555 worms treated with galangin (100 μM) or L-Dopa (2 mM) in the presence or absence of 6-OHDA (50 mM) for 72 h. Bars, S.D., *** *p* ≤ 0.001. (**D**) BZ555 worms were treated with galangin (100 μM) or NAC (5 mM) for 72 h in the presence or absence of 6-OHDA (50 mM), which was followed by incubation with DHE solution (100 µM) for 1 h. Representative images of worms were captured by a fluorescence microscope. Magnification: Scale bars: 20 µm. (**E**) The bar chart indicates the relative RFP. Bars, S.D., *** *p* ≤0.001.

To validate the neuroprotective effect of galangin in vivo, we employed the transgenic strain BZ555, in which egIs1 [dat-1p::GFP] bright GFP is observable in dopamine neuronal soma and processes [21]. As shown in Figure 7A,B, the GFP intensity in the head region of BZ555 worms, indicating the viability of DA neurons, was significantly decreased by 6-OHDA. However, supplementation with galangin or L-Dopa remarkably recovered the GFP intensity to 18.7% and 22.0%, respectively. These results suggest that galangin inhibits 6-OHDA-induced DA neurodegeneration in *C. elegans*. In addition, the 6-OHDA-treated worms exhibited a deficit in food-sensing behavior [22]. Under normal physiological conditions, worms reduce their movement and stay to feed on bacteria when they come across the bacteria lawn or food source. However, the 6-OHDA-treated worms exhibit a deficit in the food-sensing behavior, as evidenced by the decreased bending frequency of the body. As shown in Figure 7C, 6-OHDA-treated BZ555 worms failed to decrease the bending frequency with a slowing rate of 26.3% compared to the untreated BZ555 worms. After treatment with galangin or L-Dopa, the 6-OHDA-treated BZ555 worms markedly recovered their bending frequency, and the slowing rate was increased to 46.8% and 46.1%, respectively (Figure 7C). Moreover, we also measured the generation of ROS levels in worms. Figure 7D,E show that 6-OHDA-treated BZ555 worms exhibit a significant increase in the RFP intensity, suggesting that ROS are overgenerated by 6-OHDA. However, galangin or NAC treatment markedly decreased ROS levels in 6-OHDA-treated BZ555 worms. Collectively, these results indicate that galangin exhibits a neuroprotective effect in 6-OHDA-treated BZ555 worms.

## 3. Discussion

Traditional Chinese medicines (TCMs) have multicomponent, multitarget holistic benefits. Increasing studies have demonstrated that extracts or single compounds derived from TCMs exhibit potent neuroprotective effects in various models of neurodegenerative diseases [23,24,25,26]. Galangin, chemically known as 3,5,7-trihydroxyflavone, is a dietary flavonoid widely distributed in various plants such as *Alpinia officinarum, Helichrysum aureonitens, Alpinia galanga,* and propolis. These plants are classified as medicinal and food homologous in China; thus, galangin is generally considered to be safe in humans. In addition, a recent study demonstrated that the oral administration of galangin displayed no signs of toxicity in male Wistar rats with streptozotocin-induced hyperglycemia when the concentrations were up to 320 mg/kg [27]. Additionally, in male nude mice injected intraperitoneally with galangin at 30 mg/kg, there were no significant histological changes observed in the liver, renal, heart, and lung tissues, along with no alterations in the serum ALT and AST levels detected [28]. Therefore, galangin is safe and has the potential for drug development in the near future. Emerging evidence indicates that galangin exerts a variety of pharmacological activities. For example, galangin attenuates oxidative damage, inflammation, and apoptosis in a rat model of cyclophosphamide-induced hepatotoxicity by activating Nrf2 signaling [29]. Galangin ameliorates diabetic cardiomyopathy by modulating oxidative stress, inflammation, and apoptosis in rats [30]. In doxorubicin (DOX)-impaired rats, galangin significantly mitigates cognitive impairment and neurotoxicity via the NOX-1/Nrf-2/HMGB1/TLR4 and TNF-α/MAPKs/RIPK/MLKL/BDNF pathways [31]. In a middle cerebral artery occlusion (MCAO)-induced rat model of focal cerebral ischemia, galangin alleviated neurologic impairments by attenuating mitochondrial dysfunction and secondary apoptosis [32]. Neuroinflammation has been implicated in neurodegenerative diseases such as PD [33]. In the LPS-induced PD rat model, galangin significantly ameliorated microglial activation, neuronal loss, and motor dysfunction. In addition, galangin inhibited the expression of tumor necrosis factor α (TNF-α), interleukin-6 (IL-6), IL-1β, cyclooxygenase 2 (COX-2), and induced nitric oxide synthase (iNOS) via the MAPK and NF-κB signaling pathways in LPS-stimulated BV-2 microglia [34]. However, the potential therapeutic effect of galangin and its mechanism of action in PD are still not well elucidated. An increasing number of studies show that network pharmacology is a method that describes the relationship among biological processes, diseases, and drugs [35]. In this study, network pharmacology was first adopted to screen the potential core targets of galangin and PD. We collected 114 galangin-related targets, 2224 PD-related targets, and 31 common targets to construct relative networks and carried out enrichment analysis. Finally, potential core targets including HMOX1, NFE2L2, SOD1, GSR, CASP9, KEAP1, MPO, GSTP1, and CREB1, biological processes, including cell aging, apoptotic processes, cellular responses to hydrogen peroxide and hypoxia, and signaling pathways, including apoptosis, PI3K/Akt, HIF-1, TNF, and neurotrophic pathways, were obtained. Several core targets were associated with oxidative stress and apoptosis regulation, suggesting that galangin might exert an anti-PD effect via antioxidative and anti-apoptosis pathways. Recently, a molecular docking technique has emerged to predict the matching binding mode of a ligand to a macromolecular partner [36]. Therefore, we further employed molecular docking to explore the direct relationship between galangin and the core targets and found that galangin possessed a strong binding ability with NFE2L2/Nrf2, as evidenced by the high binding score. The above data indicate that galangin may activate the Nrf2 signaling pathway.

After the prediction and confirmation of core targets and signaling pathways, we further investigated the neuroprotective effect of galangin in 6-OHDA-induced PD models in vitro and in vivo. Neuronal cells are susceptible to oxidative damage due to the high content of polyunsaturated fatty acids and high consumption of oxygen [37]. Therefore, there is considerable irrefutable evidence showing that the gradual degeneration or loss of DA neurons in the substantia nigra of PD subjects, and oxidative damage and Nrf2 downregulation are important pathological mechanisms [38,39]. In this study, we employed 6-OHDA, a common PD toxin, to induce oxidative damage and apoptosis in HT22 cells and *C. elegans*. Our current experiments found that galangin significantly increased the viability of 6-OHDA-treated HT22 cells, which suggested that galangin could inhibit the degeneration or loss of neurons. The literature shows that 6-OHDA leads to mitochondrial damage, excessive generation of ROS, and the subsequent high expression of proapoptotic-related proteins [40,41]. Therefore, we further found that galangin had the capacity to inhibit ROS generation, apoptosis rate, and Bax/Bcl-2 in 6-OHDA-treated HT22 cells. This finding suggests that galangin decreases neuronal death by ameliorating damage and its resultant neuronal apoptosis. *C. elegans* are widely used in the study of aging and neurodegenerative diseases because of the following advantages: genetic conservation, well-defined neuroanatomical structure, and sophisticated genetic modification techniques [42]. The BZ555 strain, a widely studied PD model of *C. elegans*, specifically tagged the dat-1 promoter (dopamine transporter gene) with GFP in DA neurons. Our current study revealed that galangin significantly recovered the GFP intensity and improved the behavioral performance in 6-OHDA-treated BZ555 worms. In addition, the ROS assay in vivo revealed that galangin could ameliorate oxidative damage in *C. elegans*, as evidenced by the decreased RFP intensity. Taken together, galangin may be a potential drug for the treatment of PD by alleviating mitochondrial dysfunction, apoptosis, neuroinflammation, and oxidative stress.

Emerging evidence indicates that the pharmacological activation of the Nrf2/Keap1/HO-1 signaling pathway alleviates oxidative stress and is recognized as a therapeutic target of PD [43]. To explore whether the predicted Nrf2 of galangin in PD is associated with its antioxidative effect, we examined the protein expression of Nrf2, Keap1, and HO-1 by Western blotting. The results showed that galangin dose-dependently inhibited the protein expression of Keap1 and increased the protein expression of Nrf2 and HO-1. Although current data partly demonstrated that galangin activated the Nrf2/Keap1/HO-1 signaling pathway, more future experiments are needed, including the disassociation of Nrf2 from Keap1 and the nuclear translocation of Nrf2 to be supported by the activation of the Nrf2/Keap1/HO-1 signaling pathway. In addition, Nrf2 siRNA is also essential to inhibit Nrf2 expression, and we examined the neuroprotective effect of galangin in vitro and in vivo.

## 4. Materials and Methods

### 4.1. Acquisition of Potential Targets of Galangin against PD

The structural information of galangin was obtained from the PubChem database, and the SMILES format was uploaded into the SwissTargetPrediction database to identify the potential targets of galangin. Other alternative targets of galangin were obtained from the GeneCards and PharmMapper databases. The DisGeNET database and OMIM database were utilized to identify targets related to PD. The common targets of galangin and PD after duplicate removal were considered potential targets of galangin against PD. Ultimately, a Venn diagram was drawn to obtain the overlapping targets.

### 4.2. Protein–Protein Interaction (PPI) Network Construction and Screening of Core Targets

To analyze the interactions of the targets, we built the PPI network using the STRING database and entered the data into the Cytoscape_v3.7.1 software for visibility and topology analysis. The screening of core targets of galangin against PD was performed according to the following conditions: degree values are greater than or equal to twice the corresponding median; betweenness centrality and closeness centrality are greater than or equal to the corresponding median. The screening conditions were degree values greater than 8, betweenness centrality greater than 0.01815109, and closeness centrality greater than 0.5.

### 4.3. GO and KEGG Pathway Enrichment Analyses of Core Targets

The gene symbols of the common targets were input into the Database for Annotation, Visualization, and Integrated Discovery (DAVID)_v6.8 database to obtain Gene Ontology (GO) and Kyoto Encyclopedia of Genes and Genomes (KEGG) pathway analyses. The GO analysis revealed three categories for evaluating galangin action, including the genes affected by galangin in the biological process (BP), molecular function (MF), and cellular component (CC) categories. KEGG analysis was performed for target protein-related pathway screening. Visualization of GO analysis by using Cytoscape/ClueGO_v3.9.0 and GraphPad Prism v9.1.1.233 software (San Diego, CA, USA). The results of the KEGG pathway enrichment analyses were visualized as a bubble diagram using the OmicShare website.

### 4.4. Molecular Docking Simulation

Molecular docking simulation was used to investigate the binding of galangin to the core targets identified in the PPI network. The 3D structure of galangin was downloaded from the PubChem database, and the crystal structures of the core target proteins, including HMOX-1/HO-1 (PDB ID:6EHA), NFE2L2/Nrf2 (PDB ID:7K29), and KEAP1 (PDB ID:1ZGK), were obtained from the UniProt website. Sybyl-X 2.0 software (Tripos, St. Louis, MO, USA) was used for structural modification of these proteins, mainly including removing cocrystallized ligands and water molecules from the protein structures, hydrogenation, and charging [44]. Finally, we used the protocol generation technique of SYBYL to generate binding pockets and then simulated the molecular docking between galangin and core proteins. Surflex-Dock scores (total scores) denote binding affinities. The experimental databases and software used in the network pharmacology approach are shown in Table 1.

### 4.5. Materials and Reagents

The materials and reagents used in the study are listed in Table 2.

### 4.6. Cell Culture

HT22, an immortalized mouse hippocampal cell line subcloned from the HT-4 cell line, is widely used in the study of neurodegenerative diseases, such as Alzheimer’s disease and Parkinson’s disease [45]. HT22 cell line was purchased from the American Type Culture Collection (ATCC; Rockville, MD, USA). The HT22 cells were cultured in DMEM supplemented with 10% FBS, penicillin (100 U/mL), and streptomycin (100 μg/mL) in a humidified incubator at 37 °C with 5% CO_2_.

### 4.7. MTT Assay

The viability of HT22 cells was assayed by an MTT reagent as described previously [46]. Briefly, the cells seeded in 96-well plates at a density of 5 × 10^3^ cells/well were treated with the test drugs at the indicated concentrations for 24 h. After treatment, 10 μL of MTT solution (5 mg/mL) was added to the wells and incubated for 4 h. The formazan was then dissolved with DMSO. The colorimetric readings of the solutions were measured at 570 nm using a spectrophotometer (BioTek, Winooski, VT, USA). The percentage of cell viability was calculated according to the following formula: Cell viability (%) = Cells treated/Cells control × 100.

### 4.8. Hoechst 33342/PI Staining

The cell death of HT22 cells was measured by Hoechst 33,342/PI staining method. Briefly, after treatment, HT22 cells were washed 3 times with PBS and stained with 2 μg/mL Hoechst 33,342 and 2 μg/mL PI solution for 10 min. Then, the cells were washed 3 times with PBS and fixed with 4% paraformaldehyde (PFA). Representative images showing blue and red signals were captured by a fluorescence microscope and merged. The cell death of the HT22 cells was obtained by calculating the percentage of cells with PI signal to cells with Hoechst signal.

### 4.9. Cell Apoptosis Rate Analysis

The apoptosis of the HT22 cells was measured by flow cytometry using an Annexin V-FITC apoptosis detection kit (4A Biotechnology Co., Beijing, China). Briefly, after treatment, HT22 cells were collected and centrifuged at 733× *g* for 5 min. Cell pellets were then resuspended in 500 μL of 1× Annexin V solution containing FITC and PI reagents. After incubation in the dark for 15 min, cell apoptosis was analyzed by a FACSVerse flow cytometer (BD Biosciences, San Jose, CA, USA) according to the manufacturer’s instructions. Data acquisition and analysis were performed by FlowJo version 10.0 software (BD Biosciences, San Jose, CA, USA).

### 4.10. Cellular ROS Measurement

The generation of intracellular ROS levels was determined by flow cytometry using the H2DCFDA fluorescent probe. Briefly, the HT22 cells were seeded at a density of 1 × 10^5^ cells/well in 6-well plates and treated for 24 h. The cells were then collected and centrifuged at 733× *g* for 5 min. The supernatant was removed, and the cell pellet was resuspended and incubated with 5 µM H2DCFDA at 37 °C for 30 min. After incubation, the cells were centrifuged at 733× *g* and washed twice with PBS. Then, the generation of intracellular ROS in HT22 cells was analyzed by flow cytometry.

### 4.11. Western Blot Analysis

After treatment, the cells were harvested and lysed with 1× RIPA lysis buffer (Cell Signaling Technology, Beverly, MA, USA) containing EDTA-free protease inhibitor cocktail (TargetMol, Shanghai, China). The cell lysates were centrifuged at 733× *g* for 10 min at 4 °C, and the supernatant was transferred to a 1.5 mL tube. The protein concentrations were measured using Quick Start TM Bradford 1× dye reagent (Bio-Rad, Hercules, CA, USA). Then, 30 µg of protein from each sample was loaded onto an SDS–PAGE gel and separated at 120 V for 90 min. After electrophoresis, the proteins were transferred to PVDF membranes (Millipore, Darmstadt, Germany) and blocked with 10% nonfat milk for 1 h at room temperature. The membranes were then incubated overnight at 4 °C with the primary antibodies, followed by incubation with the HRP-conjugated secondary antibody for 1 h at room temperature. The bands were revealed using UltraSignalTM ECL Western Blotting Detection Reagent (4A Biotech, Beijing, China) and detected by a ChemiDoc MP Imaging System (Bio-Rad). The intensity of bands was quantified by ImageJ software (ImageJ 1.46r; National Institutes of Health, Bethesda, MD, USA).

### 4.12. C. elegans Strains, Culture, and Synchronization

The transgenic BZ555 strain [Pdat-1::GFP] was obtained from the Caenorhabditis Genetics Center (CGC). The worms were cultured at 20 °C on nematode growth medium (NGM) agar plates carrying a lawn of Escherichia coli OP50 as a food source unless otherwise stated. Synchronized eggs (embryos) were isolated from gravid adults using a bleaching solution (0.5 M NaOH and 1% NaClO) and incubated in M9 buffer at 20 °C overnight to obtain synchronized L1 larvae. Then, L4 larval worms were transferred onto NGM plates containing 5 mg/L FUDR to prevent progeny from hatching.

### 4.13. Quantitative Analysis of Dopamine (DA) Neurodegeneration

6-OHDA-treated BZ555 worms were used to induce DA neurodegeneration [47]. In brief, the synchronized L1 larval BZ555 worms were transferred onto NGM plates and cultured at 20 °C for 24 h to obtain synchronized L3 larvae. Then, the synchronized L3 BZ555 worms were incubated with 50 mM of 6-OHDA and 10 mM of ascorbic acid in S-medium blended with OP50 in the presence or absence of L-Dopa or galangin. After treatment for 1 h, the worms were washed with M9 buffer 3 times and transferred onto NGM plates containing levodopa (L-Dopa) or galangin for 24 h. Then, 5 mg/L of FUDR was added to inhibit the production of progeny. After 48 h of treatment, BZ555 worms were washed 3 times with M9 buffer and then mounted onto a glass slide with a 2% agarose pad using 100 mM sodium aside and enclosed with a coverslip. Immobilized animals were observed and photographed under a positive fluorescence microscope, and the fluorescence intensity representing the viability of DA neurons was measured by ImageJ software. At least 20 animals in each group were used, and this experiment was carried out in triplicate.

### 4.14. Food-Sensing Behavioral Test

The food-sensing behavioral test was performed in 6-OHDA-treated BZ555 worms according to our previous study [48]. In brief, synchronized L3 BZ555 worms were incubated with 50 mM of 6-OHDA and 10 mM of ascorbic acid in S-medium blended with OP50 in the presence or absence of L-Dopa or galangin for 1 h. Then the worms were washed with M9 buffer 3 times and transferred onto NGM plates containing L-Dopa or galangin. After 72 h, BZ555 worms were transferred to the center of NGM plates spotted with or without *E. coli* OP50 lawn and then allowed to recover for 90 s to avoid observing behavior caused by stress. Then, the number of body bends of the worms was counted at 20 s intervals. The slowing rate of body bending was calculated by the following formula: Slowing rate = (N without food–N with food)/N without food; N represents the total number of body bending in the presence or absence of bacteria.

### 4.15. Measurement of ROS Levels in C. elegans

The measurement of the ROS levels in the 6-OHDA-treated BZ555 worms was performed as described previously [49]. In brief, synchronized L3 BZ555 worms were incubated with 50 mM of 6-OHDA and 10 mM of ascorbic acid in S-medium blended with OP50 in the presence or absence of N-acetyl-L-cysteine (NAC) or galangin for 1 h. Then, the worms were washed with M9 buffer 3 times and transferred onto NGM plates containing NAC or galangin for 72 h. After treatment, worms were washed with M9 buffer and transferred to tubes with 1 mL of an M9 buffer containing 100 µM dihydroethidium (DHE) and incubated for 1 h in the dark. After incubation, the tubes were centrifuged at a speed of 733× *g*, and the worms were mounted onto a glass slide in an M9 medium containing 100 mM sodium aside. Representative images were captured using a positive fluorescence microscope. The DHE fluorescence intensity was quantified by ImageJ software.

### 4.16. Statistical Analysis

Statistical significance among the groups was analyzed by one-way univariate analysis of variance (ANOVA) followed by a post-hoc test using the GraphPad Prism v9.1.1.233 software (San Diego, CA, USA). *p* < 0.05 was considered to indicate a significant difference.

## 5. Conclusions

In conclusion, the current study reveals for the first time that galangin exhibits neuroprotective effects, as evidenced by the improvement of neuronal viability and behavior in 6-OHDA-treated HT22 cells and *C. elegans*, by activating the Nrf2/Keap1/HO-1 signaling pathway, which provides novel insight into the further development of galangin as a candidate for the treatment of PD in the future.

## Figures and Tables

**Table 1:** The experimental databases and software used in the network pharmacology approach.

Name	URL	Access Date
Pubchem	https://pubchem.ncbi.nlm.nih.gov/	21 January 2022
Swiss Target Prediction	http://swisstargetprediction.ch/	21 January 2022
DisGeNET	http://www.disgenet.org/	21 January 2022
OMIM	https://omim.org	21 January 2022
GeneCards	https://www.genecards.org/	21 January 2022
STRING 11.0	https://string-db.org/	24 January 2022
DAVID 6.8	https://david.ncifcrf.gov/	24 January 2022
OmicShare Tools	https://www.omicshare.com/tools/	24 January 2022
UniProt	https://www.uniprot.org	25 January 2022
Cytoscape 3.7.1	https://cytoscape.org/	12 August 2022
Sybyl-X 2.0	https://sybyl.com	25 January 2022
PharmMapper	http://www.lilab-ecust.cn/pharmmapper/	21 January 2022

**Table 2:** The materials and reagents used in the study.

Reagents and Antibodiese	Source	Identifier
6-OHDA	Sigma–Aldrich, St. Louis, MO, USA	H4381
MTT	Sigma–Aldrich, St. Louis, MO, USA	MKCD4805
5-Fluoro-2′-deoxyuridine (FUDR)	Sigma–Aldrich, St. Louis, USA	F0503
Galangin	Chengdu Desite Biological Technology Co., Ltd., Chengdu, China	DG0020-0020
Levodopa	Topscience Company, Ltd., Shanghai, China	T0848
Dulbecco’s Modified Eagle Medium (DMEM)	Gibico, Carlsbad, CA, USA	10741574
Fetal bovine serum (FBS)	Gibico, Carlsbad, CA, USA	10099141
Trypsin-EDTA solution	Gibico, Carlsbad, CA, USA	11590626
Penicillin–streptomycin solution (100×)	Gibico, Carlsbad, CA, USA	15140122
H2DCFDA fluorescence probe	Invitrogen, Carlsbad, CA, USA	D399
Annexin V-FITC/PI Apoptosis detection Kit	4A Biotech Co., Ltd., Beijing, China	20190221
Bax	CST, Beverly, MA, USA	2772
Bcl-2	CST, Beverly, MA, USA	15071S
Keap1	CST, Beverly, MA, USA	4678S
Nrf2	CST, Beverly, MA, USA	12721S
HO-1	CST, Beverly, MA, USA	70081S
β-actin	Santa Cruz Biotechnology, Dallas, TX, USA	sc-47778

## Data Availability

Data is contained within the article and Supplementary Materials.

## Supplementary Materials

The following supporting information can be downloaded at: https://www.mdpi.com/article/10.3390/ph15081014/s1, Figure S1: Full-length Western blotting images in Figure 5E and Figure 6.
