# Supplementary figures and images for: Galangin Exhibits Neuroprotective Effects in 6-OHDA-Induced Models of Parkinson’s Disease via the Nrf2/Keap1 Pathway
Source: Pharmaceuticals (Basel). 2022 Aug 17;15(8):1014. doi: 10.3390/ph15081014 (PMC9413091; doi:10.3390/ph15081014)

**Fig. 5E**

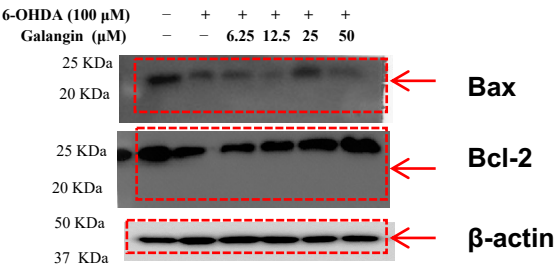

**Fig. 6A**

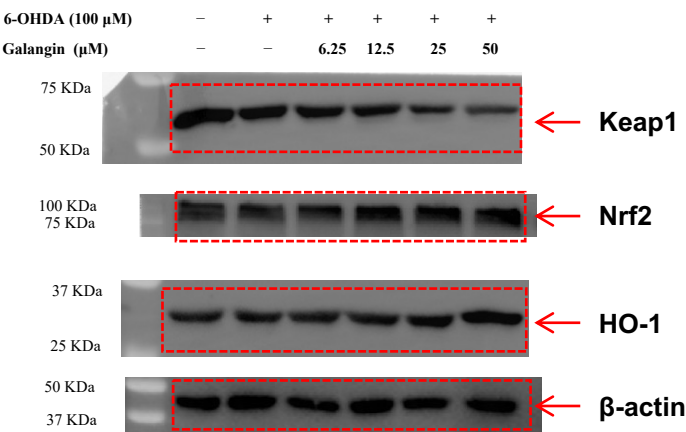

Supplement: Supplementary file 1 [file pharmaceuticals-15-01014-s001.zip › Figure S1. Full-length Western blotting images in Figures 5E and 6..pdf]
